# Local control of giant cell tumors of the long bone after aggressive curettage with and without bone cement

**DOI:** 10.1186/1471-2474-15-330

**Published:** 2014-10-02

**Authors:** Zhen-hua Gao, Jun-qiang Yin, Xian-biao Xie, Chang-ye Zou, Gang Huang, Jin wang, Jing-nan Shen

**Affiliations:** Department of Radiology, First Affiliated Hospital of Sun Yat-Sen University, Guangzhou, 510080 China; Department of Orthopaedics, First Affiliated Hospital of Sun Yat-Sen University, 58# zhongshan 2 road, Guangzhou, 510080 P.R. China

**Keywords:** Giant cell tumor of the long bone, Bone graft, Bone cement, Aggressive curettage, Local recurrence

## Abstract

**Background:**

Aggressive curettage has been well established for the treatment of giant cell tumors (GCTs) of the bone. The purpose of this study was to review our experience and evaluate the role of different implant materials in patients with GCTs of the extremities after aggressive curettage.

**Methods:**

A total of 119 patients with GCTs of the long bone were treated at the First Affiliated Hospital of Sun Yat-Sen University between 2004 and 2009. We excluded patients presenting metastases, recurrent tumors, and soft tissue involvement and those with Jaffe pathological grade III. The remaining 65 patients were treated with aggressive curettage using a bone graft or bone cement to fill the cavity. The recurrence rates and functional scores associated with the different fillings were analyzed.

**Results:**

Aggressive curettage and bone grafting was performed in 34 cases (52.3%), and aggressive curettage with bone cement was performed in 31 cases (47.7%). The overall recurrence rate after the aggressive intralesional procedures was 35.3% with bone grafting and 12.9% when bone cement was used as an adjuvant filling. The recurrence rate following aggressive curettage and bone grafting was higher than that following aggressive curettage with cement (p = 0.038). The Musculoskeletal Tumor Society (MSTS) score for bone graft patients was 91.1%, which was significantly lower than that for patients treated with bone cement (94.7%).

**Conclusions:**

The use of bone cement was associated with a significantly lower recurrence rate than bone grafting following aggressive intralesional curettage to treat benign giant cell tumors of the long bone. Better MSTS functional results were also observed in the bone cement group compared to the bone graft group.

**Electronic supplementary material:**

The online version of this article (doi:10.1186/1471-2474-15-330) contains supplementary material, which is available to authorized users.

## Background

Giant cell tumors (GCTs) are primary benign bone tumors with invasive and potentially malignant characteristics [1–3]. Intralesional curettage is the main surgical treatment option [4, 5]. After curettage, filling the cavity with bone grafts or cement is commonly performed to provide structural support and prevent collapse [6]. Previous studies have shown that using bone cement as a filler can significantly reduce the relapse rate after curettage [7–9]. In recent years, with the application of aggressive curettage technology, which is characterized by the use of a high-speed burr and other auxiliary methods, the giant cell tumor recurrence rate has been well controlled, and there is a new argument regarding the best type of implant material to use after aggressive curettage [10–12]. It is well known that the GCT outcome may differ according to many factors, including the presence of metastatic disease at diagnosis, pathological fracture, soft tissue involvement, and anatomical site [7, 13, 14]. Therefore, it is very difficult to make a reliable assessment regarding the role of different implant materials, and it is important to assess the role of different implant materials in a group of patients with the same or similar clinical conditions.

The aim of this study was to retrospectively review our experience with GCTs in patients with similar clinical conditions by assessing the contribution of different implant materials to local control and functional results.

## Methods

### Patient selection

A total of 119 patients with GCTs of the long bone were treated at the First Affiliated Hospital of Sun Yat-Sen University between 2004 and 2009. The patient selection criteria for this retrospective study were as follows: no previous treatment, no metastases at diagnosis, no pathological fracture, no soft tissue involvement, Jaffe pathological grade I or II [15], and underwent aggressive curettage. Sixty-four cases were excluded, and the remaining 65 cases constituted the group included in the current study. Then, the patients were divided into two groups according to the different local implant materials: Group 1, 34 patients who underwent aggressive curettage and bone grafting (allograft and/or autograft); and Group 2, who underwent aggressive curettage with bone cement fillings. This study was approved by First Affiliated Hospital of Sun Yat-Sen University ethics committee to access patient data for clinical research.

### Preoperative imaging and pathological examination and evaluation

The imaging procedures included preoperative anteroposterior and lateral X-ray examinations, MRI of the ipsilateral long bone using 1.5 T and 3.0 T superconductive MR units (Magnetom Vision, Magnetom Trio Tim, Siemens, Medical System, Erlangen, Germany), and a preoperative anteroposterior chest X-ray examination. Axial and coronal or sagittal T1WI (TR 420–600 ms and TE 12–20 ms) and T2WI (TR 2500–4500 ms and TE 80–120 ms) sequences were used. The scanning slice thickness was 4 mm with a 1 mm interval. Two experienced radiologists independently observed and recorded the X-ray and MRI findings of the giant cell tumors and agreed upon a diagnosis. The imaging findings included the integrity of the bone shell, with or without a soft tissue mass, and with or without lung metastases on the chest X-ray film. Histological sections and records were available in all cases and were reviewed and confirmed by two experienced pathologists.

### Tumor volume measurement

The anteroposterior and mediolateral maximum diameters of the tumors were measured on preoperative axial MR images. The longitudinal maximum diameters of tumors were measured on preoperative coronal or sagittal MR images in the long bones. The tumor volume was calculated using the formula as follows: Tumor volume = Π/6 (anteroposterior maximum diameter × mediolateral maximum diameter × longitudinal maximum diameter), according to the methods used by Bieling P et al. [16].

### Treatment protocol

**Figure 1:**
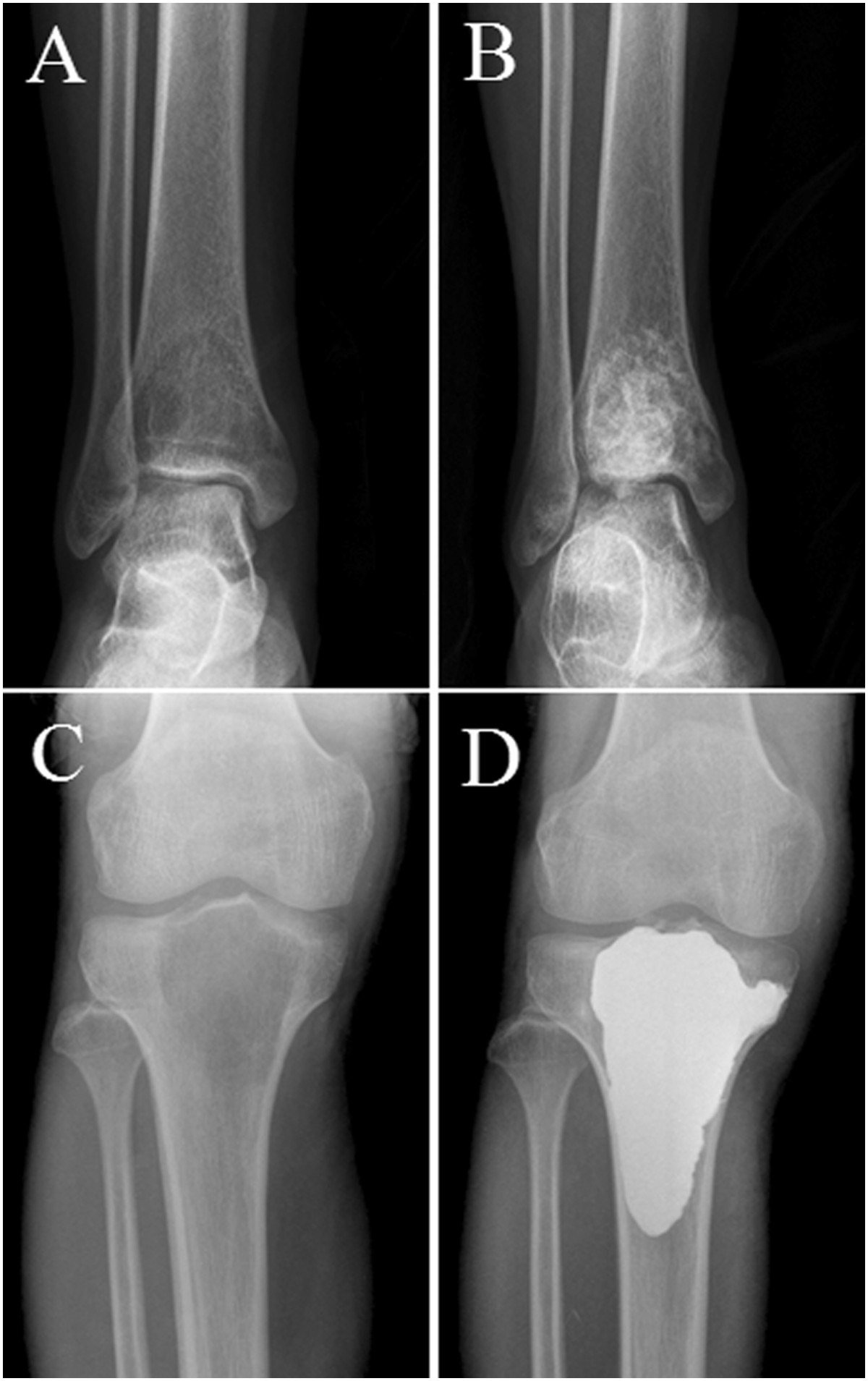
**Typical radiograph of GCT of the long bone.** Anteroposterior radiograph shows a lytic lesion in the distal tibia **(A)** and proximal tibia **(C).** Anteroposterior radiograph showing the results after agreessive curettage and filling the bone defect with bone grafts **(B)** and bone cement **(D)**.

Local treatment consisted of aggressive curettage (high-speed burring, alcohol and iodine tincture as adjuvant) and bone grafting (Figure 1B) or aggressive curettage with cement (Figure 1D). The type of local treatment was chosen for each patient based on careful consideration of data and after a discussion with radiotherapists, surgeons, and medical oncologists. The choice of local treatment was tailored to each patient’s characteristics: age; tumor site, size, and grade; and expected level of function.

### Postoperative follow-up

All patients were followed up to review clinical functional results and perform an X-ray examination at 3 months, 6 months, 9 months, and 12 months after the clinical operation and continued to be followed up every 6 months thereafter. The Musculoskeletal Tumor Society score, developed by Enneking, was used to assess functional results [17]. Local tumor recurrence was determined by X-ray, and MRI was chosen if the clinical manifestations or X-ray findings could not confirm local tumor recurrence. All recurrent tumors were confirmed by a second surgical pathology. The end of follow-up for this study was the time of tumor recurrence.

### Statistical analysis

The chi-square test was used to evaluate the statistical association between two variables. The Kaplan–Meier and log-rank methods were used to draw and evaluate the significance of event-free survival curves. One-way ANOVA was used to highlight different functional results between the different implant materials. A difference was considered statistically significant when the P-value was less than 0.05. Statistical analyses were performed using the Statistical Package for the Social Sciences (SPSS), version 19.0 (SPSS Inc., Chicago, USA).

## Results

### Clinical appearance

**Figure 2:**
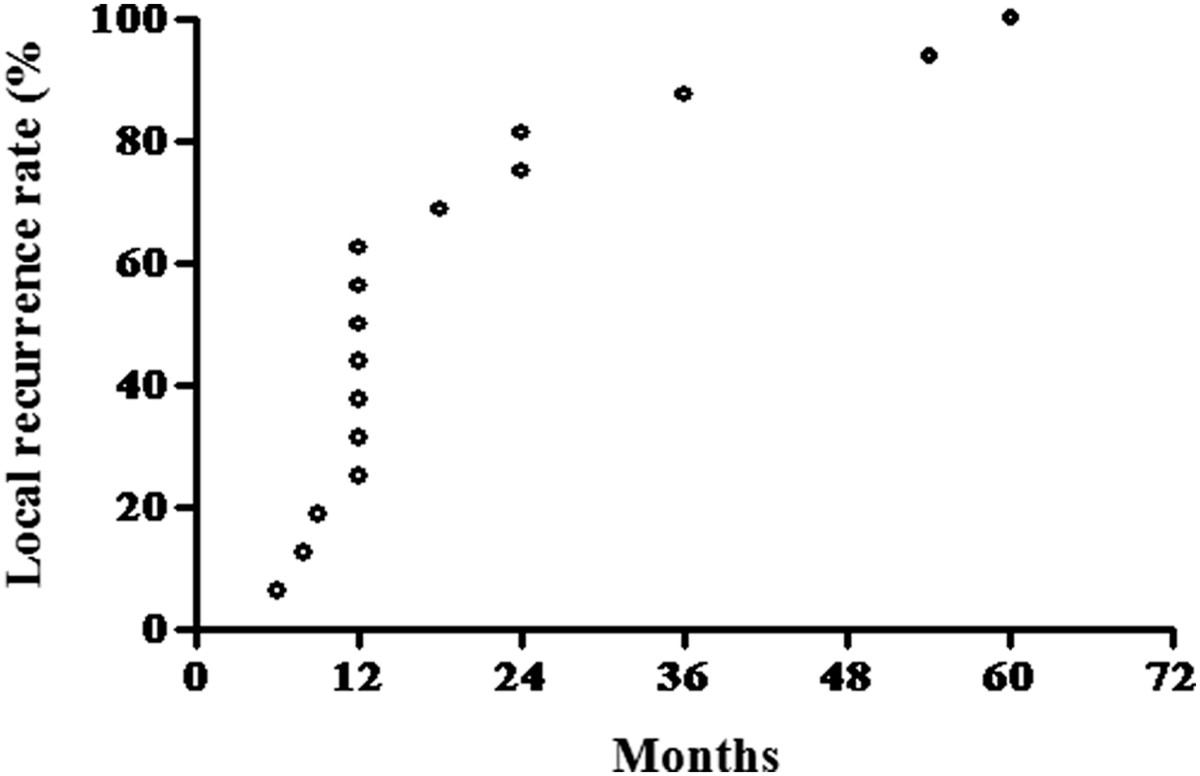
**The interval between surgery and local recurrence for the 16 recurrene patients treated at our hospital.**

Of the 65 patients in our study, 33 were male and 32 were female, with a mean age of 31.8 years (range: 18–65 years). The tumor site was the proximal femur in 4 cases, the distal femur in 28 cases, the proximal tibia in 27 cases, the distal radius in 5 cases, and the distal tibia in 1 case. The tumor volume ranged from 4 to 310 ml, with an average of 68 ml. The Jaffe pathological grades of the tumors were as follows: I, 14 cases; II, 51 cases. The mean follow-up time was 38.8 months, ranging from 6 to 84 months. At the scheduled follow-up visits, 49 patients (75.4%) had no evidence of disease, and 16 (24.6%) demonstrated local recurrence. The interval between surgery and local recurrence for the 16 patients treated at our hospital ranged from 6 months to 5 years (average, 19.8 months) postoperatively. Thirteen patients (81.3%) had a local recurrence within 2 years after surgery (Figure 2). Only 3 patients (18.7%) had a recurrence after more than 2 years.

### Event-free survival analysis

**Figure 3:**
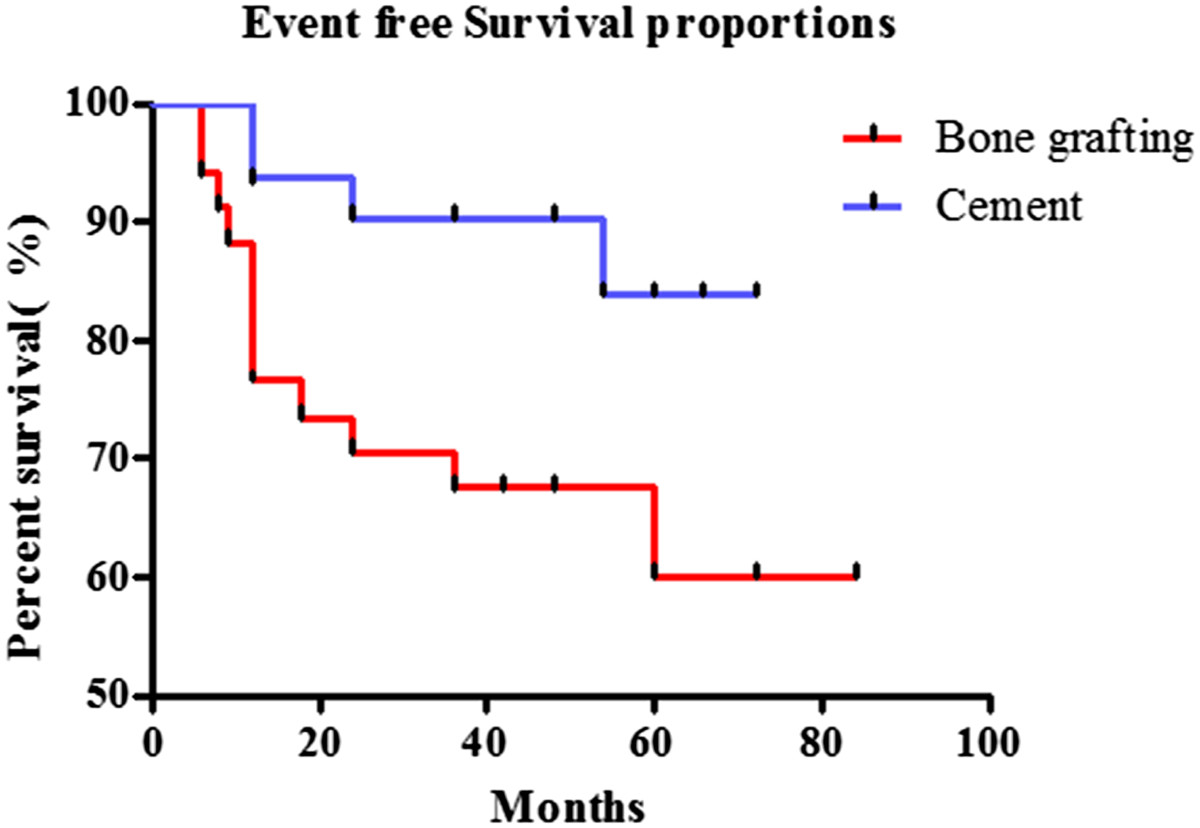
**Kaplan–Meier life table analysis of cumulative event free survival survival of GCT patients according to different local treatment (p = 0.0264).**

Univariate analyses, as shown in Table 1, were first performed for all 65 patients. Sex, age, tumor site, tumor volume, and pathological grade were not significant. The rate of 3-year EFS was significantly lower for local treatment with bone grafting compared to cement (64.7% vs. 87%, *P* = 0.038). In contrast to other studies, proximal femur cases had the best prognosis (100%, 3-year EFS), and the distal femur location had the worst (71.4%, 3-year EFS). Even more interestingly, patients with a tumor volume less than 50 ml had a worse 3-year EFS (71.0%) than those with larger tumors (79.4%); this difference was not significant (P = 0.433). Although patients with Jaffe pathological grade I had a higher 3-year EFS than those of grade II, this difference was not significant (P = 0.089). The Kaplan–Meier and log-rank life table analyses also confirmed that local treatment with cement was significantly associated with a higher probability of better events and a better outcome (Figure 3).

**Table 1:** **Analysis of clinic factors predicting 3-year event free survival (EFS) of 65 patients**

Variable	No.of cases (65)	EFS patients (49)	% EFS (75.4)	P-value
**Sex**				0.944
male	33	25	75.8	
female	32	24	75.0	
**Age(Yrs.)**				0.616
≤30	33	24	72.7	
>30	32	25	78.1	
**Site**				0.75
Proximal femur	4	4	100.0	
Distal femur	28	20	71.4	
Proximal tibia	27	20	74.1	
Distal radius	5	4	80.0	
Distal tibia	1	1	100.0	
**Tumor volume(ml)**				0.433
≤50	31	22	71.0	
>50	34	27	79.4	
**Grade**				0.089
I	14	13	92.9	
II	51	36	70.6	
**Local treatment**				0.038
Bone graft	34	22	64.7	
Cement	31	27	87.1

### Comparison of different adjuvant therapies

To further investigate the effects of different adjuvant therapies for local control, we compared the clinical features of different local treatments (Table 2). Of the 34 patients in the progressive curettage and bone grafting group (Group 1), 17 were male and 17 were female. The age ranged from 18 to 49 years old, with an average of 30.7 years. The tumor site was the proximal femur in 4 cases, the distal femur in 11 cases, the proximal tibia in 13 cases, the distal radius in 5 cases, and the distal tibia in 1 case. The tumor volume ranged from 4 to 185 ml, with an average of 42 ml. The Jaffe pathological grades were as follows: I, 10 cases; II, 24 cases. The follow-up time was between 6 and 84 months, with an average of 37.7 months. Local tumor recurrence was evident in 12 patients in this group (35.3%). The tumor was detected from 6 to 60 months (mean 17.9 months) after local treatment.

**Table 2:** **Comparison of clinic data of 65 patients with GCT according to different treatment group 1 vs group 2**

Patient data	Group 1	Group 2	P-value
No.of patients	34	31	
**Sex**			0.897
Male	17	16	
Female	17	15	
**Age (Yrs.)**			0.716
≤30	18	15	
>30	16	16	
**Site**			0.358
Proximal femur	4	0	
Distal femur	11	17	
Proximal tibia	13	14	
Distal radius	5	0	
Distal tibia	1	0	
**Tumor volume(ml)**			<0.001
≤50	26	5	
>50	8	26	
**Grade**			0.109
I	10	4	
II	24	27	
**Local recurrence**			0.038
Yes	12	4	
No	22	27	

Of the 31 patients in the progressive curettage and cement group (Group 2), 16 were male and 15 were female. The age ranged from 19 to 65 years old, with an average of 33.1 years. The tumor site was the proximal femur in 1 case, the distal femur in 16 cases, and the proximal tibia in 14 cases. The tumor volume ranged from 28 to 310 ml, with an average of 96 ml. The Jaffe pathological grades were as follows: I, 4 cases; II, 27 cases. The follow-up period ranged from 12 to 72 months, with an average of 46.5 months. In this group, local recurrence was detected in four patients (12.9%) at 12 to 54 months (mean 20.4 months) after surgery.

Regarding the patient clinical features of the two groups, one significant difference was tumor volume (Table 2). In the bone graft group, tumor volume was significantly smaller compared to the cement group (P < 0.001). The local recurrence rate of the bone graft group was also higher compared to the cement group, and this difference reached statistical significance (P < 0.05).

### Different MRI manifestation in the two groups

**Figure 4:**
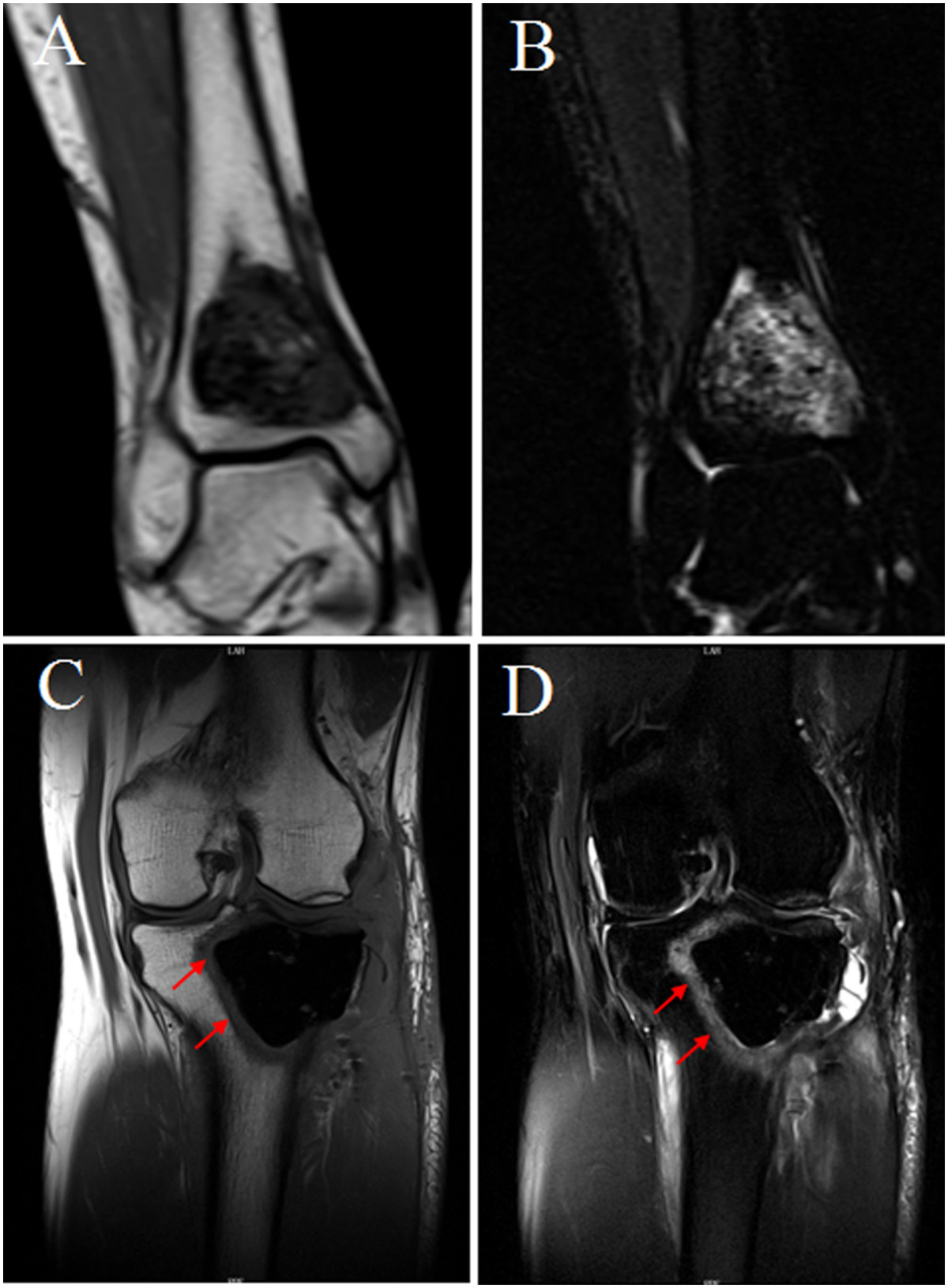
**Different MRI findings between bone grafts and bone cement group after agreessive curettage during follow up.** The coronal T_1_WI **(A)** and coronal fat-suppressed T_2_WI **(B)** showed that the band signal around the area filled with bone cement. No similar MRI findings in the bone grafting group **(C and D)**.

Six patients have been underwent MRI examination during follow up with an average of 9.8 months (6–14 months), 4 in the bone graft group and 2 in the cement group. An abnormal banded signal around the area filled with bone cement was found in the two cement group cases (Figure 4A,B). Howerer, there was no similar MRI findings in the bone grafting group (Figure 4C,D).

### Comparison of the MSTS functional scores in the two groups

Better functional results were observed following treatment with cement compared to bone grafting. The mean score in the cement group was 94.7 (SD 5.4), whereas it was 91.1 (SD 7.5) after bone grafting. One-way ANOVA showed a significant difference between the type of adjuvant therapy and functional, outcome (P = 0.011).

## Discussion

GCTs of the bone are aggressive and potentially malignant primary bone tumors that often occur at the end of the long bone in adults aged 20 ~ 40 years old [18, 19]. These tumors are primarily composed of stromal cells and multinucleated giant cells; the stromal cells are the main tumor cell component in GCTs of the bone [20, 21]. According to microscopic morphological findings, Jaffe created a pathological classification system for GCTs, including grades I-III. In the new bone tumor classification released by the WHO in 2002, GCTs of the bone were divided into GCTs and malignant GCTs. The former are equivalent to grades I ~ II, and the latter is equivalent to grade III.

Surgical treatment options include intralesional excision or segmental resection [7, 14]. Curettage has a higher recurrence rate [22, 23], but it preserves adjacent joint function. The ideal treatment of GCTs consists of excising the tumor and sparing the joint. Therefore, many scholars believe that GCTs should be treated using curettage [24, 25]. To avoid local recurrence, aggressive curettage has been widely used and has achieved good clinical results [12, 14]. The recommended aggressive curettage technique involves opening the bone through a large cortical window that allows visualization of the entire tumor cavity. After curettage is achieved, the cavity is deepened with the use of high-speed burrs [14, 26]. Various adjuvant therapies (including phenol and liquid nitrogen) may be employed in conjunction with curettage, and these most likely reduce the risk of recurrence compared with curettage alone [19, 25].

After tumor evacuation, the cavity can be filled with cement or bone grafting [10]. The literature is divided as to whether the bone defect should be filled using bone grafts or cement. In the Scandinavian Sarcoma Group multicenter study by Kivioja et al. [8], which involved 294 patients, filling the cavity with cement was shown to be a prognostic factor. The recurrence rate was 20% for filling with cement and 56% for intralesional surgery without cementation (p = 0.001). Becker WT et al. [13] has also reported that the use of bone cement as an adjuvant significantly reduces the recurrence rate following intralesional treatment of benign giant cell tumors, and it appears to be the therapy of choice for primary as well as recurrent giant cell tumors of the bone. By contrast, in the Canadian multicenter study by Turcotte et al. [11], which involved 186 patients, the adjuvant method or filling material was not significantly associated with the risk of recurrence. By retrospectively reviewing the records and images of 621 extremity GCT patients between 1989 and 2009, Niu X et al. [12] also concluded that bone grafting did not affect local tumor control after aggressive curettage and that the local recurrence rate was 11.1% if bone grafting was used. Similar results were also reported by Errani C et al. [14]; although cement decreased local recurrence, the influence of adjuvants was not statistically significant.

Many factors might influence the treatment outcome of GCTs [7, 13, 27], and none of these studies were randomized. Therefore, the evaluation of prognostic factors and the assessment of different local treatments may be affected by selection bias. Errani C [14] stated that no prospective randomized studies have shown the effects of different methods of filling the cavity. However, the main shortcoming of these retrospective reviews is the analysis of patients over a long time period, over which many changes in imaging studies, pathological examinations, and surgical treatments occurred, altering the diagnostic approach and treatment of patients with GCTs.

To avoid this problem, we used detailed patient selection criteria for this retrospective study performed for 2004–2009, including patients who underwent aggressive curettage. The operative procedure was also limited to three senior surgeons in our department. Our retrospective review attempted to identify prognostic factors useful to evaluate the risk level for each patient and possibly determine the strategies of GCT treatment.

After the univariate analysis, no significant statistical effect on the local recurrence rate was observed for gender, age, tumor volume, or Jaffe grade. Only the type of implant materials emerged as a significant factor. Bone cement was shown to be more effective at treating GCT compared with bone grafting. The Kaplan–Meier and log-rank life table analysis also confirmed that local cement treatment was significantly associated with a higher probability of better events and a better outcome. Regarding our patients’ clinical features, the only significant difference between the two groups was tumor volume (Table 2); in the bone grafting group, tumor volume was significantly smaller compared to the cement group (*P* < 0.001). Patients with small primary tumors might have a better prognosis and be more likely to be cured by bone grafting. However, bone grafting patients with smaller tumors relapsed more often compared to the cement group, which further suggests that bone cement is an effective adjuvant to treat GCT of the long bone, even for larger tumors.

Similar to marginal excision, it is difficult to completely remove residual tumor cells in the inner wall of the cavity using aggressive curettage, and there is still the possibility of relapse. Compared with bone grafting, bone cement can be combined with firmly scraping the edges of the residual cavity. When bone cement solidifies, it releases polymerizing heat reaching 80-90°C, which has a high-temperature inactivation effect on the residual cavity of the tumor [28, 29]. These factors are most likely the main reasons for the lower recurrence rate with bone cement fillings compared to bone grafting. We also found an abnormal banded signal around the area filled with bone cement by MRI (Figure 4A,B), which could reflect damage to the surrounding bone marrow due to the high-temperature effect. Howerer, there was no similar MRI findings in the bone grafting group (Figure 4C,D).

In most cases, the tumor recurred during the first two years (81.3%) after surgery in our series, which is consistent with other studies [1, 14], but the longest recurrence required 5 years to develop. Therefore, we suggest that patients should be evaluated through at least the 5th year after the final surgery. The data also showed that the GCT recurrence rates were 66.7% for bone grafting and 50% for bone cement one year after surgery, while they were 83.3% for bone grafting and 75% for bone cement in the first two years. These data indicate that the different implants and different postoperative times may lead to differences in the tumor recurrence rate following aggressive curettage of giant cell tumors of the long bone.

In other studies, tumor location significantly affected prognosis. Due to the difficulty associated with treatment, the distal radius and proximal femur are associated with a higher rate of local recurrence [14, 30]. However, there was no statistical correlation between tumor location and prognosis in our series.

For giant cell tumors of the long bones, the theoretical advantages of bone grafting, if the tumor does not relapse, include the ability of autologous or allograft bone to achieve bone healing, satisfactory recovery and no revisions. In contrast to our expectation, better functional results were observed in the cement group compared to the bone grafting group (P = 0.011) in our series. This discrepancy may due to the early weight-bearing activities of cement group patients and the short follow-up period during which bone cement-related complications, such as osteoporosis, were observed less often.

The limitations of our study include the retrospective analysis and the lack of random assignment of the type implant material used due to the tailored choice made according to each patient’s requirements.

## Conclusion

In conclusion, we showed that the use of bone cement in a group of patients with GCTs of the long bone resulted in a lower local recurrence rate when compared to bone graft patients following aggressive intralesional curettage treatment. Better MSTS functional results were also observed after bone cement compared to the bone graft group. A prospective randomized study evaluating the effects of different methods of filling the cavity should be performed in the near future.

## Authors’ original submitted files for images

Below are the links to the authors’ original submitted files for images. Authors’ original file for figure 1 Authors’ original file for figure 2 Authors’ original file for figure 3 Authors’ original file for figure 4

## Abbreviations

GCTs: Giant cell tumors
MRI: Magnetic resonance imaging
SD: Standard deviation
MSTS: Musculoskeletal Tumor Society
WHO: World Health Organization.
