# Supplementary figures and images for: Local control of giant cell tumors of the long bone after aggressive curettage with and without bone cement
Source: BMC Musculoskelet Disord. 2014 Oct 2;15:330. doi: 10.1186/1471-2474-15-330 (PMC4196200; doi:10.1186/1471-2474-15-330)

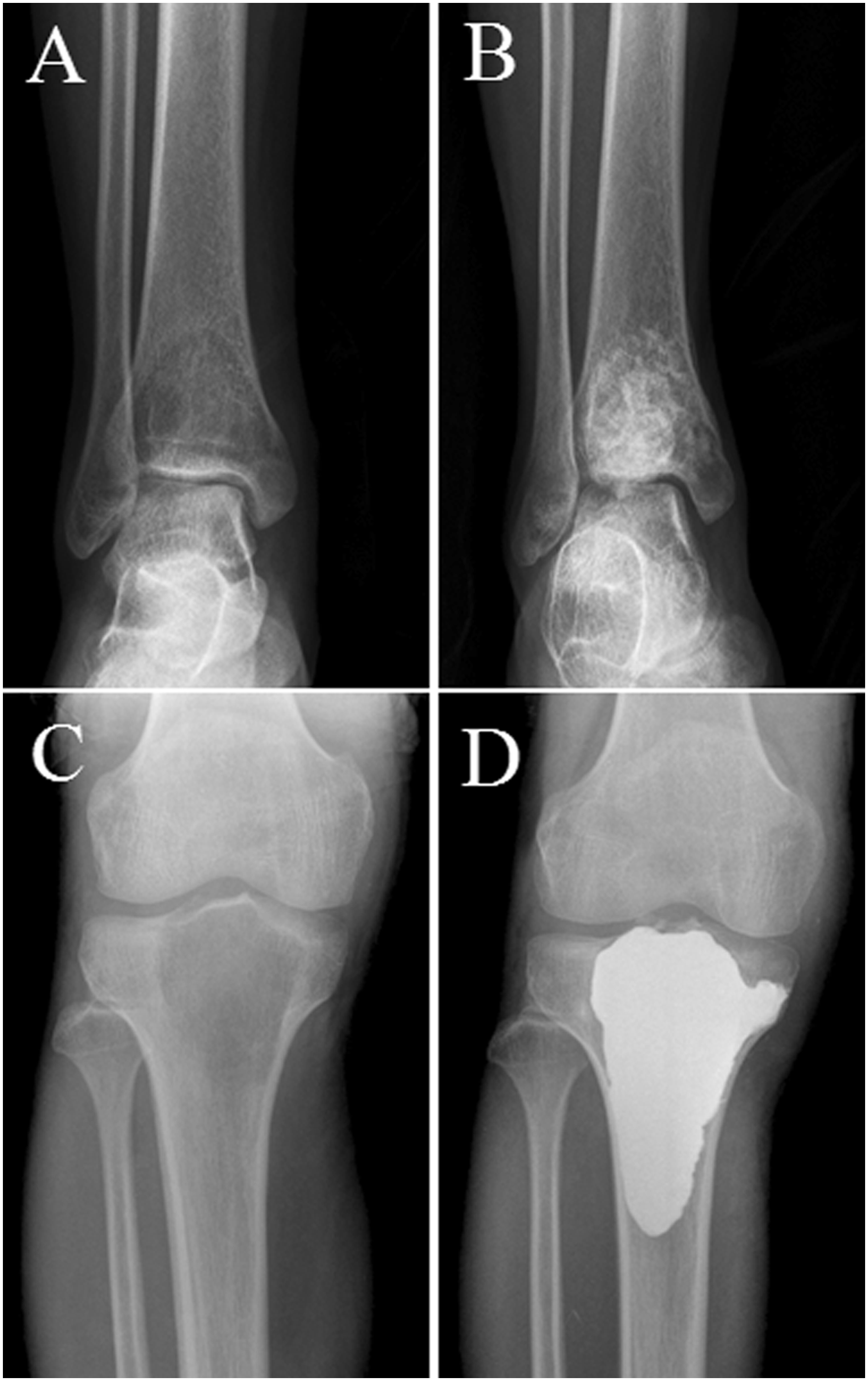

Supplement: Supplementary file 1 — Authors’ original file for figure 1 [file 12891_2014_2270_MOESM1_ESM.tif]

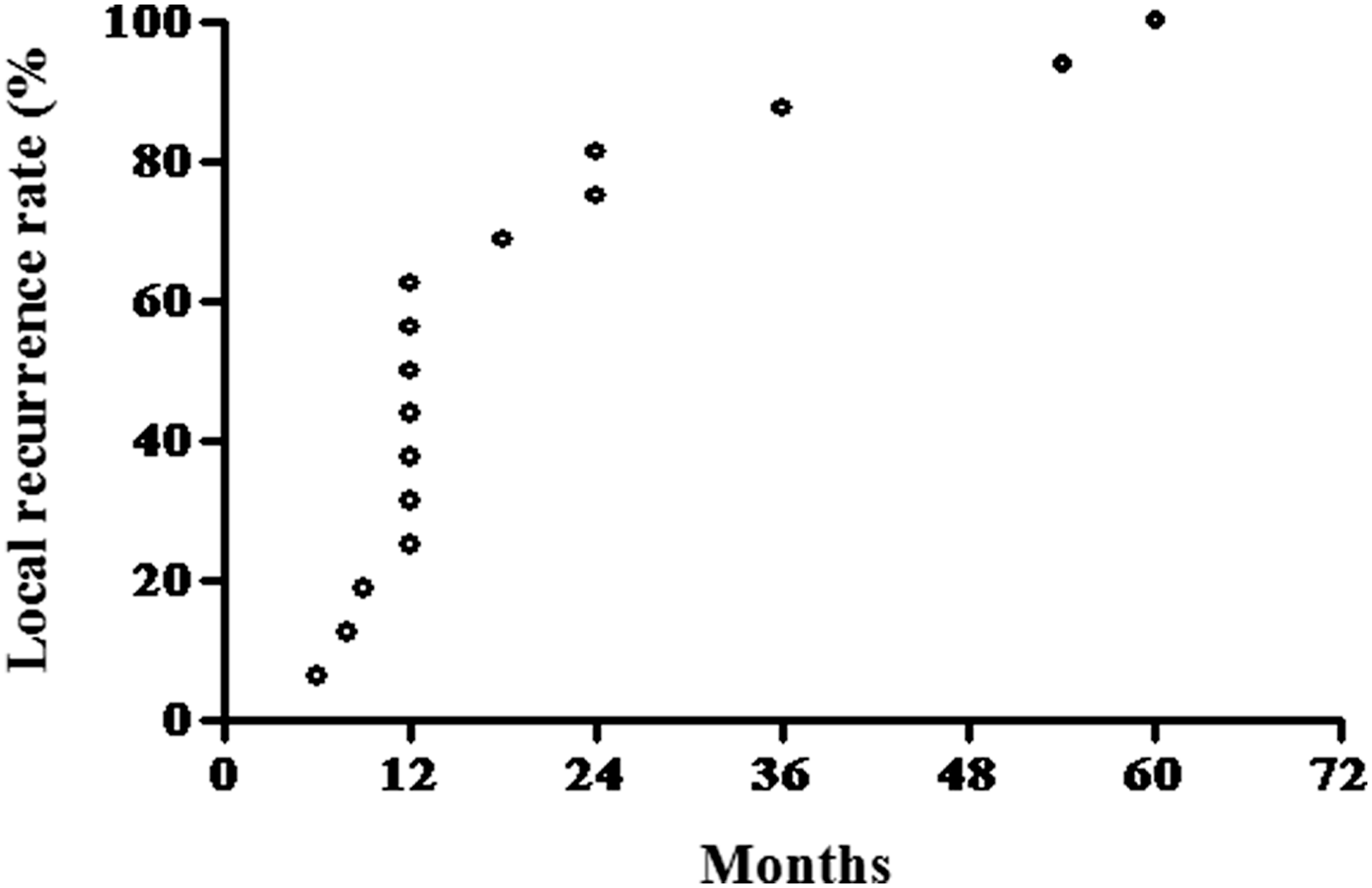

Supplement: Supplementary file 2 — Authors’ original file for figure 2 [file 12891_2014_2270_MOESM2_ESM.tif]

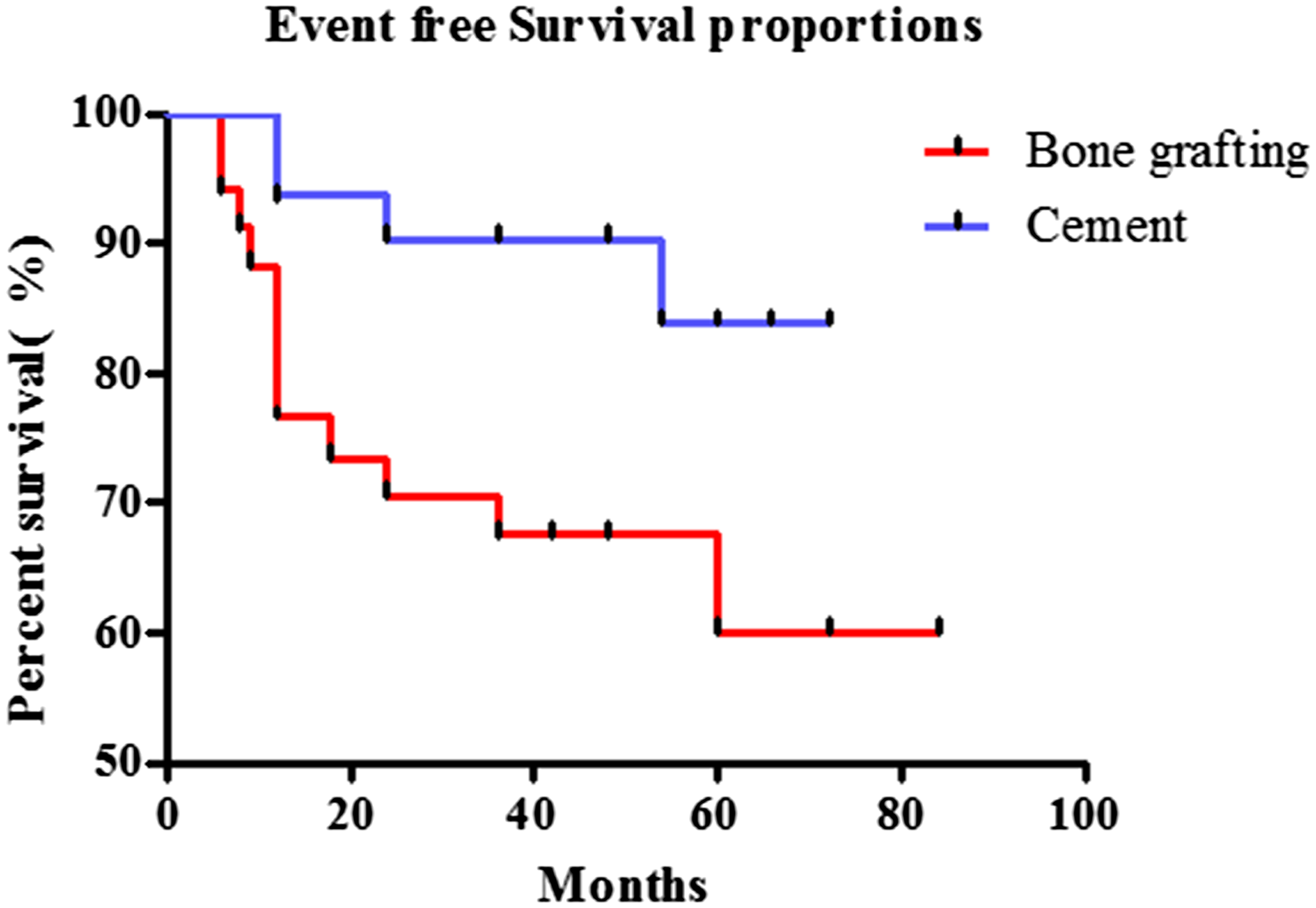

Supplement: Supplementary file 3 — Authors’ original file for figure 3 [file 12891_2014_2270_MOESM3_ESM.tif]

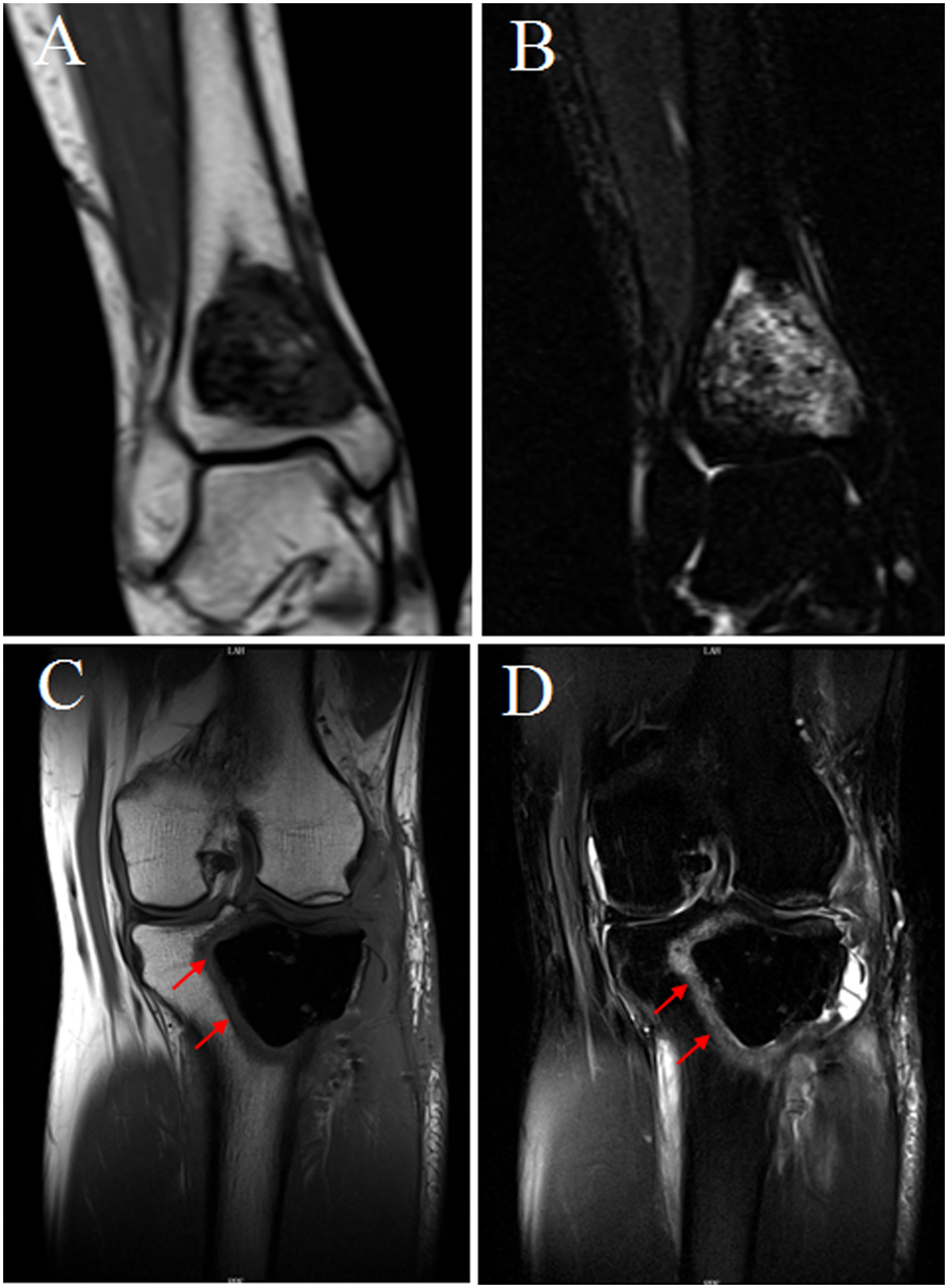

Supplement: Supplementary file 4 — Authors’ original file for figure 4 [file 12891_2014_2270_MOESM4_ESM.tif]
